# Patients’ and healthcare professionals’ experiences and perceptions of physiotherapy services in the emergency department: a qualitative systematic review

**DOI:** 10.1186/s12245-018-0201-z

**Published:** 2018-10-11

**Authors:** Rosalie Barrett, Louise Terry

**Affiliations:** 1 School of Health and Social Care, London South Bank University, 103 Borough Road, London, SE1 0AA UK; 2 London South Bank University, London, UK

**Keywords:** Physiotherapy, Emergency department, Perceptions, Experiences, Systematic review

## Abstract

**Background:**

Worldwide, emergency department (ED) attendances and admissions to acute care have increased significantly. Many EDs are adding physiotherapists to their team thereby allowing doctors to see more cases that are ‘urgent’. This is a move away from the ‘traditional’ physiotherapy service whereby the ED team refers patients to an outpatient physiotherapy service sometimes resulting in significant delays. Internationally, there is no agreed consensus on the role or value of ED-based physiotherapists.

**Aim:**

The objective of this review was to retrieve, critically appraise and synthesise the evidence from studies relating to patients’ and healthcare professionals’ experiences and/or perceptions of physiotherapy services in the ED.

**Method:**

This is a systematic review (SR) synthesising qualitative studies, which have considered patients’ (population 1) and healthcare professionals’ (population 2) experiences and/or perceptions (outcomes) of ED physiotherapy services (exposure). A comprehensive systematic search, limited to English language articles, was undertaken on seven electronic medical databases (Medline, EMBASE, CINAHL, AMED, BNI, PubMed and PEDro) for the period January 2006 to October 2016. Grey literature was identified using Google Scholar, reference lists and website searching. The Critical Appraisal Skills Programme (CASP) qualitative checklist was used to appraise all included studies. All studies were data extracted and quality appraised by two reviewers to enhance rigour and reduce bias.

**Results:**

A total of 2163 studies were screened, 10 received full-text review and 7 studies were included in the final review. Six of the studies originated in Australia and one from the USA. The themes that emerged were as follows:

- Patients and healthcare professionals view ED-based physiotherapists as having (1) expert clinical skills and (2) an educational role.
- There is role confusion and lack of integration of the ED-based physiotherapist within the ED team.

**Conclusion:**

This review adds an in-depth human perspective to the current ED physiotherapy literature, which provides insight into how ED healthcare services and physiotherapy services specifically should be developed and delivered in the future. The knowledge from this review has implications for future education programmes, as well as development of both new care pathways and physiotherapy clinical roles.

Research into ED-based physiotherapy services is predominantly quantitative. Despite the newness of the ED physiotherapy role, this review reveals that the provision of physiotherapists within EDs contributes value to both patients and staff. However, the dominance of Australian research means it is uncertain how it translates to the UK or elsewhere. There needs to be further UK-based research.

## Review

### Background

In the last decade, emergency department (ED) attendances and admissions to acute care have increased significantly. The physiotherapist skill set is not just restricted to the expert treatment of musculoskeletal (MSK) conditions [1] but it plays roles in managing complexity, such as assessing, treating and managing patients with a wide range of clinical presentations including falls and neurological and respiratory conditions. Some physiotherapists who have undertaken advanced practice training may also be able to suture, plaster and prescribe, for example, allowing these practitioners to autonomously treat minor injuries and enabling them to provide a “one-stop-shop” approach to care. Consequently, physiotherapists are being appointed to EDs, urgent care centres and minor injury units worldwide allowing doctors to see patients who require more immediate input [2–4]. An ED physiotherapist has been defined as a ‘clinician dedicated to working as a member of the ED team to manage patients either autonomously or in conjunction with other attending medical or nursing staff’ [5]. This shifts EDs from ‘traditional’ teams comprising of doctors and nurses where physiotherapy was previously a ‘referred to’ service [6].

Internationally, literature examining ED physiotherapy has considered the impact services have on waiting times, safety, costs and access [1, 7, 8] and their effectiveness in treating MSK conditions and achieving patient satisfaction [9, 10]. Quantitative research dominates as confirmed by Kilner’s systematic review SR [2]. Although some qualitative research has considered in-depth views of patients’ and healthcare professionals’ experiences and perceptions of ED physiotherapy services currently, there is no agreed consensus on the role or value of ED-based physiotherapists. The aim of this SR was to retrieve, critically appraise and synthesise the evidence from studies relating to the perceptions and experiences of patients and emergency department practitioners.

## Methods

### Search strategy

A comprehensive systematic search, limited to English language articles, was undertaken on seven electronic medical databases (Medline, EMBASE, CINAHL, AMED, BNI, PubMed and PEDro) for the period January 2006 to October 2016. Grey literature was identified using Google Scholar, reference lists and website searching. Search terms included are in Table 1. Boolean indicators, wildcards and truncations were used as appropriate.

**Table 1:** PEO, search terms and key words, inclusion and exclusion criteria

PEO component	Search terms/key words	Inclusion criteria	Exclusion criteria
Population 1 Patient	Patient, service user, client	Primary research, qualitative research, peer-reviewed journal articles and grey literature, research related to the adult population, published between January 2006 and September 2016, English language only, UK and International studies	Systematic reviews, categorical data, standardised questionnaires with no text response
Population 2 Healthcare profession	Physiotherapist, physiotherapy, physical therapist, healthcare profession, clinician, practitioner, extended scope practitioner, advanced practitioner		
Exposure Physiotherapy services in the ED	Emergency department, accident and emergency, emergency care, prehospital care		
Outcomes Experience and perceptions	Experiences, interviews, perceptions, qualitative, qualitative research		

## Article selection

**Fig. 1:**
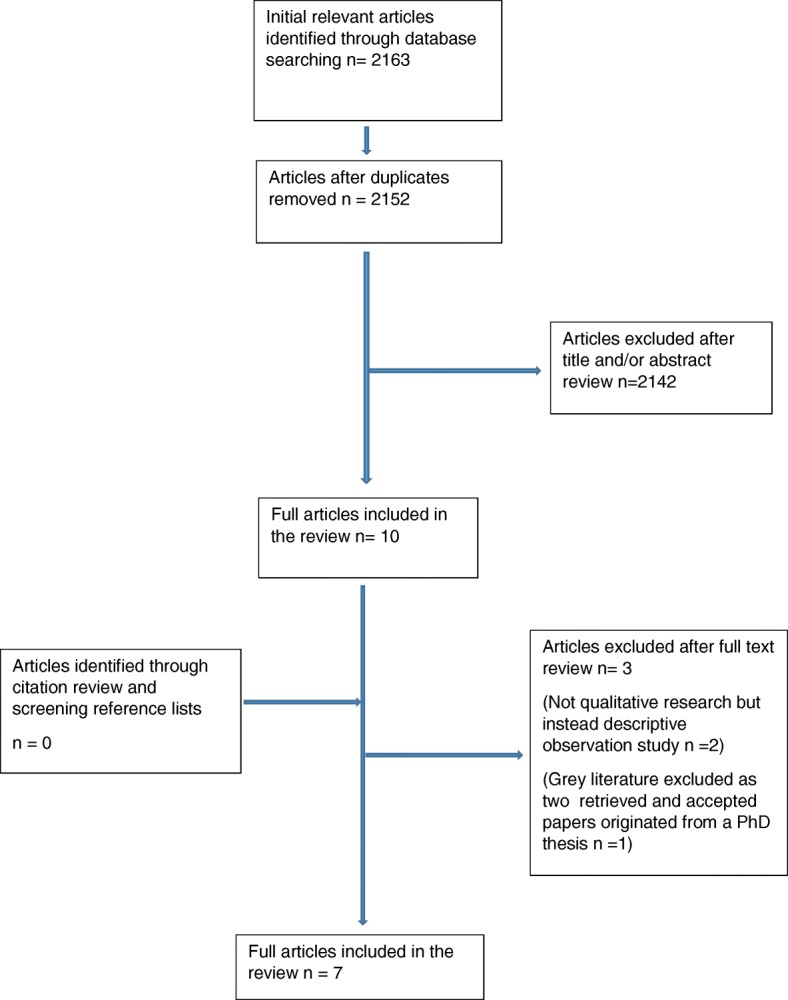
PRISMA diagram

In total, 2163 studies were found. Studies were selected for review that met the inclusion criteria. The article selection process is illustrated in the PRISMA diagram (Fig. 1) and the selected articles in Table 3. One doctoral thesis and seven peer-reviewed studies were initially selected to be included in this SR. On further examination, two of the articles included were reports from findings of this thesis and therefore excluded. Three studies [6, 11, 12] dealt specifically with healthcare professionals’ experiences and perceptions of physiotherapy ED services and three [13–15] looked specifically at patients’ experiences and perceptions. One mixed method study [16] considered both patients and health professionals’ perceptions and experiences. The qualitative data from this study was included in this review as it was the only study to explore both population groups. Kilner and Sheppard’s study [6] collected data using an Internet-based survey primarily employing categorical data but had nine textual-response questions which were thematically analysed so this study was included for the data from the open-text questions.

Sandelowksi and Barroso’s Typology of Qualitative Findings was used to categorise articles (Table 2) and confirm their appropriateness for inclusion since studies may be labelled qualitative research but lack appropriate methodology and simply produce qualitative results [17].

**Table 2:** Classification of included studies based on Sandelowski and Barroso’s typology [17]

Classification	Number of studies	Author
No findings	0	
Topical survey	0	
Thematic survey	2	Kilner and Sheppard (2010) [6] Anaf and Sheppard (2010) [13]
Conceptual/thematic description	3	Lebec et al. (2010) [11] Morris, Vine and Grimmer (2015) [16] Harding et al. (2015) [14]
Interpretive explanation	2	Lefmann and Sheppard (2014) [12] Sheppard, Anaf and Gordon, (2010) [15]

## Data extraction and analysis

Data extraction for each article was conducted by two people allowing comparison for consistency aiding inter-rater reliability [18]. Extracted data included: demographics, study aims, participants, sample size, methods and conceptual or thematic findings (Table 3).

**Table 3:** Studies included in this systematic review and summary characteristics

Authors	Title	Participants, sample selection	Method	Setting	Findings	Strengths/weaknesses of Study
Anaf and Sheppard, (2010) [13]	Lost in translation? How patients perceive the extended scope of physiotherapy in the emergency department	Patients attending ED, total *n* = 80 Metropolitan ED Melbourne *n* = 40 Regional ED Queensland *n* = 40 Adults	Qualitative questionnaire, open questions on opinions of ED physiotherapist, role of physio-therapist, suggestions for ED service improvements, demographics. Interpretative thematic analysis	Australia	Key themes: • Skills of physiotherapists • Translating physiotherapist role into ED • Selected ED physiotherapist practice Lack of agreement between participants at the two centres as to the role of the physiotherapist in ED	Questionnaire piloted for dependability and trustworthiness. Ethical issues addressed fully
Harding et al. (2015) [14]	Patient experience of expanded-scope-of-practice musculo-skeletal physio-therapy in the emergency department: a qualitative study	Patients attending ED, total *n* = 16 Metropolitan hospital *n* = 9 Rural setting *n* = 16	Descriptive observational study. One-to-one semi-structured interviews conducted some days after discharge from ED. In the case of the metropolitan hospital these were by phone but in person, at the rural hospital. Thematic analysis	Australia	Themes: • Patient satisfaction • Personal attributes of ED physiotherapists • Confidence in ED physiotherapist skills • Timing and efficiency of ED physiotherapy service	Calls it a descriptive observational study but it was simply descriptive qualitative. Refers to data triangulation but in fact it was just using more than one person to carry out thematic analysis. Not acknowledged time delay from ED visit to interview nor the fact interviews data were collected differently at the two sites
Kilner and Sheppard, (2010) [6]	The ‘lone ranger’: a descriptive study of physio-therapy practice in Australian emergency departments	Physiotherapists working in ED *n* = 28. Snowballing recruitment strategy	Descriptive cross-sectional study. 28 *Q* = categorical data and frequency analysis 9 *Q* = open text, thematic analysis	Australia	Descriptive demographic data relating to context of physiotherapists within ED. Themes related to: • Roles and role confusion • Role development • Discharge planning • Education	Piloted the questionnaire
Lebec et al., (2010) [11]	Emergency department physical therapist service: a pilot study examining physician perceptions	ED Physicians *n* = 11	Descriptive qualitative study. Interviews thematically analysed	USA	Themes: • Value of ED-based physiotherapy • Challenges of ED physiotherapist service • ED physiotherapist characteristics	Pilot study – no evidence of full study being undertaken. Not clear if the interviews were one-to-one or group. Interview tool provided
Lefmann and Sheppard, (2014) [12]	Perceptions of emergency department staff of the role of physiotherapists in the system: a qualitative investigation	ED doctors *n* = 2 Nurses *n* = 2, Physio-therapists *n* = 2	Individual interviews, thematically analysed	Australia	Themes: • Clinical skills of ED physiotherapists • Balancing autonomy with collaboration within ED team • Preserving the professional self	Very small number of participants so not really possible to draw conclusions regarding differences between professions. However, this study is part of a doctoral study (Anaf/Lefmann) with *n* = 80 participants. Participants checked their transcripts. Ethical procedures not discussed
Morris, Vine and Grimmer, (2015) [16]	Evaluation of performance quality of an advanced scope physiotherapy role in a hospital emergency department	Quantitative evaluation of patients attending ED over 11 months Qualitative interviews Patients *n* = 11 Staff *n* =?	Prospective 53-week observational pilot study. Essentially, a service evaluation. Random selection of patients interviewed by telephone. Purposive sampling of staff. Weak form of content analysis used for interview data	Australia	Findings reported related to: • Service availability and patient throughput • Compliance with national targets • Doctors considered ED physio service safe • Nurses valued physio clinical expertise • Patient satisfaction with service	Questions for the interview are given. Many of them are closed questions and some could be interpreted as leading. • Does not state how many staff interviews were conducted
Sheppard, Anaf and Gordon, (2010) [15]	Patient satisfaction with physiotherapy in the emergency department	Patients treated by a single Melbourne ED-based physio-therapist, purposeful sampling, convenient recruitment. (*n* = 22)	Qualitative interpretative design. Face to face interviews (*n* = 22) followed by telephone interviews 2–3 weeks later (*n* = 15). Thematic analysis supported by reflexive journal	Australia	Themes related to: • Patient expectations • Bedside manner of physio • Physiotherapy management • Patient satisfaction	A fourth paper from the Sheppard team. Lacks generalisablity as this is more like a 360-degree appraisal of one person than research

The Critical Appraisal Skills Programme (CASP) [19] quality assessment tool for qualitative studies was used to appraise studies for credibility, integrity and trustworthiness.

An adapted version of the analytic process outlined by Sandelowski and Barroso was used to aid data synthesis [17]. Findings were extracted from articles. Convergence, divergence and patterns were noted. Findings were coded and thematic-networking undertaken [20, 21] to identify and ascribe the initial categories of themes. Multiple themes were ascribed initially before the final thematic categories emerged. All stages were completed simultaneously and not sequentially. As specific studies in this review considered either patients’ or healthcare professionals’ experiences and/or perceptions of physiotherapy services in the ED, separate analyses were undertaken for each population group and convergent themes were then merged.

## Results

### Quality of studies

None of the studies in this review were excluded following the appraisal process; however, there was variation in their quality. All were checked to see whether they had obtained approval from a relevant Research Ethics Committee (this checkpoint is also part of the CASP criteria). Six studies confirmed they were granted approval; one study failed to explicitly document this [11]. This does question the trustworthiness of the research [22], and by omitting this, it is difficult to determine whether there were any conflicts of interest. The majority of the studies did document the procedures undertaken to ensure ethical issues were considered, such as confidentially and informed consent [6, 12–14]. However, not all acknowledged ethical considerations, which does limit applicability. Trustworthiness was considered [6, 13, 15], for example, data collection methods were piloted before commencing the final study to ensure that the chosen participant questions were appropriate to elicit full in-depth responses. Kilner and Sheppard’s study lacked a truly qualitative feel due to its reliance upon an internet-based survey [6]. Harding et al.’s study was described as an observational study but only interviews were reported, no observations [14]. Small sample sizes were a problem in the case of Lefman and Sheppard’s [12] and Morris et al.’s [16] who only identify three staff participants.

Two studies in this SR [12, 14] and several in a previous SR [23] reported undertaking triangulation which aids the credibility of their findings [24]. One study reported undertaking multiple-analyst triangulation, where four researchers were involved [14]. Sheppard et al. also commendably acknowledged that the researcher undertook writing a reflexive diary [15]. This process can enhance research triangulation further and improve the overall rigour of qualitative findings [25]. However, this researcher appeared to be the only data analyst. A final consideration, which can enhance the integrity of qualitative findings, is member checking [26]. Two studies [11, 12] undertook this process.

### Geographical location

Six of seven studies in this review were undertaken in Australia [6, 12–16], and therefore, the application of these findings to other international physiotherapy ED posts is limited. The experiences and perceptions of ED physiotherapy services are likely to vary from country to country and even from department to department within the same country due to demographic differences and influencers [27]. Furthermore, the same researchers dominate this subject field. Anaf has authored two of the studies in this review [13, 15] and Sheppard four [6, 12, 13, 15], biasing the findings further.

### Themes

The themes that emerged showing agreement by patients and healthcare professionals were that ED-based physiotherapists have (1) expert clinical skills and (2) an educational role. A third theme, (3) being part of the ED Team, is related to role confusion and a view that there is a lack of integration and belonging of the ED-based physiotherapist within the ED multidisciplinary team (MDT). These themes are discussed below in a narrative synthesis.

1. Expert clinical skills

Patients across all studies perceived physiotherapists to be clinical specialists. There was variation in their experiences and perceptions. Physiotherapists were viewed as specialists in rehabilitation post injury [13]. Not all patients were explicit that these skills were a ‘specialisation’ of a physiotherapist in the ED, instead some patients perceived these skills to be those they would expect to find from any ED clinician [13].

There was a general consensus that ED physiotherapists are specialists in acute MSK assessment and management. Patients viewed physiotherapists as experts in MSK management [13–16], and some were happy to see a physiotherapist instead of a doctor [14]. Other patients referred to physiotherapist’s MSK specialism by explaining their experiences of treatment with a physiotherapist; for example, they talked about physiotherapists as practical professionals, providing hands-on treatment, exercises and functional assessments [13, 15].

All healthcare professionals in Lefmann and Sheppard’s study [12] valued the ability of ED physiotherapists to undertake a thorough MSK assessment and offer a specialist service for patients. Doctors also perceived this, saying “they are the experts” [11, p6]. Physiotherapists and doctors agreed that ED physiotherapists require enhanced MSK knowledge and appropriate skill to work in the ED [6, 11]. Advanced nurses perceived that ED physiotherapists had MSK expertise and even perceived their skill to be more advanced than that of ED doctors [16]. Doctors acknowledged that an MSK physiotherapy intervention combined with medical input allowed for an accurate diagnosis and thorough treatment strategy that was beyond ‘the norm’ of their ED [11]. Nurses and doctors felt that having MSK expertise improved quality of care and satisfaction for patients in the ED [11, 16].

Beyond the MSK expertise, all studies identified that patients viewed the ED physiotherapist as having a broad spectrum of clinical skills. Patients perceived physiotherapists to be very thorough clinicians [14–16] and felt that their assessments were thoroughly covering wide aspects of care, including social history, environment, and functional status [15]. Patients also viewed ED physiotherapists as being able to problem-solve and recognised their contribution in treating and managing pain, respiratory conditions and effectively treating the elderly [13, 15]. Patients had confidence in the clinical ability of ED physiotherapists [13] and were aware of their scope of practice; they were also satisfied that physiotherapists knew when to refer on [14].

Although physiotherapists themselves reported that, as well as being skilled in MSK conditions, they also had generic clinical skills [12], doctors and nurses in the same study did not, however, share this view, only discussing the MSK contribution physiotherapists brought to the ED ([12]. In a different study, physiotherapists agreed, reporting that their skill was ‘generic’ and that they also had additional training in occupational therapy and social work; however, this was the only study to mention specific cross-professional skill [6]. Physiotherapists perceived their care in the ED to be holistic [12]. Both doctors and physiotherapists acknowledged that physiotherapists were able to appropriately treat vestibular disorders, providing an alternative option for patients in the ED and accelerating patient care [11, 12]. Physiotherapists and doctors also perceived ED physiotherapists as having appropriate skills to treat elderly patients in the ED, undertake mobility and safety assessments [6, 11, 12] and wound care [11]—this was the only study that discusses this—although physiotherapists did express they would like to be trained in suturing [6]. Doctors and physiotherapists reported that ED physiotherapists had advanced or ‘extended scope’ skills in X-rays [6, 16] and plastering; however, there was no consensus amongst physiotherapists as to whether these skills were considered as ‘extended scope’ [6].

Overall, both patients and healthcare professionals perceived physiotherapists to be comprehensive holistic clinicians, exploring all aspects of healthcare, and able to competently treat a wide variety of conditions, including respiratory illnesses, vertigo and wound care.

2. Educational role

A valued aspect of the ED-based physiotherapist was their ability to inform, educate and advise. Patients perceived ED physiotherapists as being professional with enhanced communication skills [13–16] and that they were also empathetic, supportive and encouraging which aided patient confidence in coping with their injury [13, 14]. Patients reported receiving education and management strategies about how to manage their condition [13–16]. Patients recounted receiving advice on movement, safety and mobility aids and perceived physiotherapists to be valuable in developing their confidence to self-manage [15].

All studies including healthcare professionals showed that the ED physiotherapist was perceived as an “educator” contributing to patient and/or colleague education. Lebec et al.’s study of ED doctors found that physiotherapists were valued for offering extensive patient education which played a crucial role in preventing later complication or developing chronic illness [11]. Doctors reported asking for advice about referrals to outpatient services and expressed an interest in working alongside physiotherapists when treating patients with vertigo or skin wounds [11] or MSK injury [11, 16]. Nurses also perceived physiotherapists’ knowledge as a useful education opportunity [16]. Physiotherapists felt they contributed to educating the ED MDT on MSK management [6]. Doctors and physiotherapists also acknowledged that physiotherapists had a responsibility to educate the ED MDT about their clinical expertise to ensure physiotherapists are accepted into the team and that their skills are utilised appropriately [6, 11].

3. Being part of the ED team

Healthcare professionals perceived that physiotherapy ED services were beneficial, but perceptions around how they achieved this varied. Physiotherapists felt they offered timely and efficient MSK assessment and treatment in the ED and were key in discharge planning and onward referral to ensure timely community or hospital care [6, 12]. Doctors and nurses agreed, saying that immediate physiotherapy input benefited onward continuity of care and recovery [11, 16]. Physiotherapy ED presence positively influenced the speed of ED care for others as the MDT was freed to see other cases [11]. This was especially beneficial from a nursing perspective when the ED was busy [12, 16]. However, some doctors in Lebec et al.’s study [11] believed that additional ED MDT members slowed patient turnover although they acknowledged that the time spent was crucial to patient care. Some doctors reported only understanding the benefits of an ED physiotherapy service after they had worked with ED physiotherapists [11]. Doctors were the only professional group that expressed that physiotherapists in the ED had unique personal characteristics, reporting that they enjoyed working alongside them. Physiotherapists and doctors expressed a requirement for physiotherapists to be educated to a specific clinical level to work in the ED environment [6, 12]. The physiotherapy ED service holds benefits for both staff and patients, and some doctors and nurses suggested that the hours of service should be extended [16].

The evidence base from patients was less clear as to the value of physiotherapists in ED. Some patients reported that they were unaware that their consultation had been with a physiotherapist, presuming instead the physiotherapist was a doctor [16]. Patients also reported that they had not expected to be treated by a physiotherapist in the ED [13, 15]. Patients who had previously experienced ED physiotherapy care expected to see a physiotherapist [15] particularly for an MSK issue [14].

There were variations in the level of patient satisfaction. Some patients suggested that the physiotherapy service was the best aspect of their ED experience [15] and others simply described a positive experience [14] or reported that it was adequate [15]. Positive satisfaction was expressed in relation to the organisation and speed of ED physiotherapy care [14, 16] and of follow-up care [13–15]. Some patients were not satisfied with seeing a physiotherapist in the ED and expressed that they would have preferred to have seen a doctor or a nurse [14]; however, other patients described their experience as “thorough” and “better than expected” [14].

There were varying views amongst healthcare professionals as to whether physiotherapists were working autonomously, but still as part of the ED MDT, or in complete isolation. Doctors viewed physiotherapists as part of the MDT, and acknowledged that they wished to work alongside physiotherapists to aid their own learning and were even happy for physiotherapists to question their own MSK diagnoses and for them to undertake the initial assessment [11, 12]. Doctors did express, however, that this was only once they trusted the physiotherapist’s clinical expertise [11, 12]. Physiotherapists reported that it was challenging to simultaneously try and enhance their clinical autonomy in the ED, while also trying to be accepted into the wider ED team. To overcome this, physiotherapists acknowledged that it was key to build strong relationships with medical colleagues and demonstrate clinical competence to aid trust [12]. Physiotherapists also felt they were always having to prove themselves in the ED and sought additional support beyond the ED environment from their wider physiotherapy network to avoid feeling professionally isolated [6, 12].

There was no conclusion as to whether an ED physiotherapy service provided an opportunity or professional challenge. Healthcare professionals acknowledged that ED physiotherapy remains an “unrecognised” aspect of ED care due to the poor understanding about what physiotherapists can offer patients and their clinical expertise [11, 12]. Doctors and nurses expressed caution over physiotherapists taking on advanced roles in the ED [12]. Nurses articulated concern that physiotherapists may be asked to treat some of their caseload [12]. However, some doctors perceived this new ED profession as healthy competition between the MDT [12]. Physiotherapists generally felt confident in their clinical ability but recognised there could be resistance to their presence from ED colleagues and perceived this as a reluctance to change and embrace new roles in an environment that has always been medically dominated [12]. Physiotherapists had divided views over whether their ED role should be seen as an opportunity to extend their scope of practice and professional boundaries, or whether they should simply work within their own remit and respect the expertise of other professionals [6].

## Discussion

Patients and healthcare professionals perceived physiotherapists to have a wide-ranging skill set. Both population groups considered physiotherapists to be experts in MSK management, with the majority of patients feeling comfortable being seen by physiotherapists in the ED for MSK injury. Recent evidence has found that physiotherapists are recognised as MSK experts and appropriately treat this clinical group in the ED [28]. There were, however, variances between the two population groups about the specific additional clinical skills attributed to ED physiotherapists. Overall, both patients and healthcare professionals agreed that physiotherapy ED intervention is not just isolated to MSK care. Wider research reports that ED physiotherapists have a role in discharge planning, assessing mobility and falls and treating patients with respiratory and neurological conditions [1, 29–32]. A recent opinion piece by Lefmann and Crane [27] openly discusses the wide-ranging skill mix and varying roles that ED physiotherapists have. This perceived variation of ED physiotherapy skill, may be because individual ED physiotherapy services are likely to be influenced by local (and national) demographics, health concerns and political and economic factors [33], and therefore, the clinical remit of physiotherapists across any ED department is likely to vary [27]. Furthermore, what is acceptable for a physiotherapist to undertake in one country may be considered outside the scope of practice for another [27].

Patients and healthcare professionals acknowledged that physiotherapists play a role in education. Patients valued the breadth of information they were provided about their injury and after-care, including how to self-manage once leaving the ED. Health professionals valued the professional education they gained from working with ED physiotherapists, specifically recognising their MSK expertise and contribution to patient care. Research has confirmed that ED education by a physiotherapist plays a key role in patient self-management [34] and can reduce further complication, falls risk and prevent re-admissions to the ED [35].

Interestingly, physiotherapists were the only population group to express that working in the ED provided them with the opportunity to develop further clinical expertise. Literature has confirmed that physiotherapists working in the ED do have additional clinical skill [5] such as ordering and interpreting diagnostic tests [36, 37] or (in the UK specifically) independently prescribing [29]. Patients and other healthcare professionals did not acknowledge this development opportunity; however, this is not surprising as professional development is likely to be more important to the specific professional group in question.

Physiotherapists openly reported that feeling accepted into the ED MDT was a struggle. They felt that they needed to prove their ability to be accepted and acknowledged. They also missed peer support and had to seek this elsewhere. Both doctors and nurses did express initial wariness of ED physiotherapists, and these perceptions do offer an explanation as to why physiotherapists felt isolated. However, once the physiotherapy service was established and doctors were aware of the physiotherapists’ input and competence, they perceived them to be part of the larger ED MDT. This does hint that perhaps physiotherapists were accurate in their perception that they needed to ‘prove themselves’ within the department.

Healthcare professionals perceived physiotherapy services to improve access to care for patients, by offering timely treatment, by organising onward referral or by freeing up other ED MDT members to see patients. These findings have been supported by the wider literature which has identified that ED physiotherapy services can help improve patient flow within the ED [2–4], provide shorter waiting and treatment times and facilitate quicker discharges compared to other clinical practitioners [7].

The physiotherapy ED role in the ED MDT is a new and emerging discipline [31] compared to the established ED doctor and nurse. Considering this, it is not surprising that patients within these studies did not expect to see a physiotherapist, or at times, were unaware that their consultation was undertaken by a physiotherapist. This could be a result of a number of factors. For example, did a busy environment contribute to information being lost? Did the ED physiotherapist fail to appropriately communicate their job role? Is there a lack of awareness that a physiotherapist may work in ED, and therefore, this role was not expected by the public? Alternatively, is there confusion around healthcare professional titles and what these mean to patients and the public? Anaf and Sheppard’s study acknowledged this confusion, entitling their study “lost in translation”, suggesting that what patients perceive ED physiotherapy to be is not necessarily reflected in actual practice [13]. The limited evidence in this review does not offer a conclusive answer; however, this finding does raise ethical issues (informed consent) around patient care that future research needs to address.

Healthcare professionals acknowledged confusion around the understanding of the remit and role of physiotherapy ED services and reported that their understanding developed only after working with these services. As discussed, this may be because ED physiotherapy is still a new discipline, and until there is sufficient, quality research confirming the contribution physiotherapy services make to the ED, it is likely that this uncertainty will continue [7, 27]. The research, which focuses heavily on MSK physiotherapy roles, has attempted to discuss what the ED physiotherapy role might be and what their extended scope skills are; however, there is variation in the understanding on both fronts [2, 27]. As discussed, local and national political and economic influencers [16] are likely to have led the development of these posts, rather than credible research, and this explanation is supported by the fact there are currently no set guidelines, competencies or agreed job descriptions for these ED posts [27].

### Limitations

The studies included had methodological limitations, limiting the credibility of this review. Many of the studies that undertook interviews had very small sample sizes, and the majority only undertook their research in one ED for a limited timeframe, making it challenging for the findings to be applicable to larger populations [38]. Future studies need to consider including a larger variation of population groups across more ED sites, this would build on the themes generated in this review and enhance understanding of patient and healthcare professionals’ experiences and perceptions of ED physiotherapy services. This review also included a prospective observational study which collected a variety of data [16], as it was the only study to provide data for both population groups although the qualitative data provided was minimal. The research is dominated by Australia and by two researchers in particular so transferability to other healthcare systems is limited. Separate, independent quality appraisal and data extraction of each article included, with subsequent discussion to achieve consensus, reduced the potential for bias to occur.

## Conclusions

To the authors’ knowledge, this is the first qualitative SR to explore the perceptions and experiences of patients’ and healthcare professionals’ of ED physiotherapy services. It adds a unique in-depth human perspective to the current ED physiotherapy literature, acknowledging that perception and experience can provide valuable insights into how new ED healthcare services should be developed and delivered. The ED physiotherapy role remains poorly understood, and the exposure of this through this review identifies that the physiotherapy profession needs to consider how it raises awareness about the skill set physiotherapists can offer and how physiotherapy markets itself, both to the general public (patients) and to ED healthcare practitioners, to ensure their expertise is acknowledged and used appropriately. Understanding the patient experience is paramount to delivering ED care, this SR provides valuable knowledge, which may have useful implications for physiotherapy practice, education (pre and post graduate), new and current physiotherapy services and ED healthcare professionals. The evidence and research for this physiotherapy role is scarce; however notwithstanding this, new posts continue to be developed [27], suggesting that service development is perhaps ahead of clinical research and patients’ understanding of the role of the ED physiotherapist. This review demonstrates that there is a knowledge gap in relation to this emerging role, particularly in countries outside Australia and the USA. Despite the newness of this role and the fact that its qualities are relatively unknown, the provision of physiotherapists within emergency departments contributes value to both patients and staff.

## Abbreviations

CASP: Critical Appraisal Skills Programme
ED: Emergency department
MDT: Multidisciplinary team
MSK: Musculoskeletal
SR: Systematic review
